# Heart rate recovery is useful for evaluating the recovery of exercise tolerance in patients with heart failure and atrial fibrillation

**DOI:** 10.1007/s00380-021-01839-6

**Published:** 2021-03-30

**Authors:** Seiya Tanaka, Taro Miyamoto, Yusuke Mori, Takashi Harada, Hiromi Tasaki

**Affiliations:** 1 Department of Cardiovascular Medicine, Kitakyushu Municipal Yahata Hospital, 2-6-2 Ogura, Yahatahigashi-ku, Kitakyushu, Fukuoka 805-8534 Japan; 2 Department of Internal Medicine, Kitakyushu Municipal Yahata Hospital, 2-6-2 Ogura, Yahatahigashi-ku, Kitakyushu, Fukuoka 805-8534 Japan

**Keywords:** Heart rate recovery, Heart failure, Atrial fibrillation, Cardiac rehabilitation

## Abstract

This study aimed to examine the factors that contribute to improvement of exercise tolerance in patients with heart failure (HF) and atrial fibrillation (AF) following cardiac rehabilitation. Our hypothesis is that parasympathetic values are important for recovering exercise tolerance in those patients. We included 84 consecutive patients with HF and AF (mean age: 69 ± 15 years, 80% men). All of the patients underwent a cardiopulmonary exercise test and had pre and post 5 month cardiac rehabilitation assessed. After 155 ± 11 days and 44 ± 8 sessions, 73 patients (86%) showed an increase in peak oxygen uptake (*V*O_2_) and *V*O_2_ at the anaerobic threshold. In univariate linear regression analysis, the % change in heart rate recovery, plasma B-type natriuretic peptide levels, resting heart rate, and the minute ventilation /carbon dioxide output slope were significantly related to that of peak *V*O_2_ (*p* < 0.01, *p* = 0.03, *p* = 0.02, *p* < 0.01, respectively). Stepwise multivariate linear regression analysis showed that the % change in heart rate recovery was independently related to that of peak *V*O_2_ (*p* < 0.05). Our results suggest that heart rate recovery is closely associated with recovery of exercise tolerance in patients with HF and AF after CR.

## Introduction

Atrial fibrillation (AF) is a major cardiac rhythm disturbance that is frequently encountered in clinical practice. Even though AF is common in patients with underlying cardiac disease, it also occurs in those without cardiac disease and its incidence is increasing yearly. Risk factors for developing AF include age, sex, obesity, and a low fitness level. Current management of AF mainly focuses on rate and rhythm control and reducing the risk of stroke, and its associated morbidity and mortality [1, 2]. Rate control therapy, including heart rate control and anticoagulant therapy, is unable to improve AF-derived exertional dyspnea. However, development of new therapeutic strategies against AF, including catheter ablation, has improved symptoms of these patients [3].

The benefit of cardiac rehabilitation (CR) programs has been shown in many cardiac diseases, including patients with AF [4–7]. The mechanisms by which exercise improves health outcomes for patients with AF include atrial remodeling, antiarrhythmic effects via changes in autonomic control, reduced blood pressure, reduced body weight, and reduced lipids [8].

In patients with heart failure (HF), worsening of myocardial function is accompanied by the appearance of neurohormonal derangement, with sympathetic activation. Autonomic responsiveness is important in HF and AF. HF and AF have been mechanistically linked to changes, especially in cardiac vagal control. CR can affect sympathovagal balance and reactivity in patients with HF [9]. We hypothesized that parasympathetic values are essential factors in recovery of exercise tolerance of patients with HF and AF.

## Methods

### Participants

A retrospective analysis of data collected from 245 in to outpatients with HF was performed. The recruitment continued from October 2014 to 2017. HF was diagnosed by a cardiovascular specialist according to the European Cardiology Society HF guidelines [10]. Patients with AF comprised those with an AF rhythm at the time of a cardiopulmonary exercise test (CPX) during 5 months (*n* = 84). Persistent AF was defined as continuous AF lasting longer than 7 days. Long-standing persistent AF was defined as continuous AF lasting for longer than 1 year. Informed consent regarding CPX was obtained from all patients. All patients were followed during a 5 month CR program with education on medication and lifestyle. The exclusion criteria were as follows: paroxysmal AF; symptomatic HF; acute coronary syndrome; and an implanted pacemaker, inability to achieve an adequate pedal rotation speed, or a maximal respiratory exchange ratio of < 1.05 during CPX. The institutional ethical review board at Kitakyushu Municipal Yahata Hospital approved the study protocol. All patients provided written informed consent.

### CPX

Before training, we determined the exercise capacity of each patient by symptom-limited CPX on a bicycle ergometer. To stabilize respiratory exchange, patients were asked to remain still on the ergometer for at least 4 min before starting exercise. After a 4 min warm-up with a fixed workload, a ramp protocol of 10 W/min was started and continued until exhaustion, which was defined as shortness of breath and leg fatigue, or signs of myocardial ischemia. The protocol included a 1 min cool-down period with no added workload at the end. Then they sat down for 4 min with continued monitoring of blood pressure and heart rate. We used 12-lead continuous electrocardiographic monitoring during the test and cuff blood pressure/ was recorded manually every 2 min. Oxygen uptake (*V*O_2_), carbon dioxide output (*V*CO_2_), minute ventilation (*V*_E_), the fraction of end-tidal O_2_, and the fraction of end-tidal CO_2_ were measured on a breath-by-breath basis by computerized metabolic monitoring (Cpex-1; Inter Reha Co., Ltd., Tokyo, Japan). Peak *V*O_2_ was recorded as the mean value during the last 20 s of the test and is expressed in ml kg^−1^ min^−1^. The peak respiratory exchange ratio (RER: ratio of *V*CO_2_/*V*O_2_ at peak exercise) was used as a measure of the patient’s effort during testing and a value > 1.05 was considered to be sufficient as an objective index [11]. Borg Scale [6–20] > 17 was also considered as a subjective index.

The ventilatory anaerobic threshold point was primarily determined using the *V*-slope method [12] in addition to the following conventional criteria [13, 14]: *V*_E_/*V*O_2_ increases after registering as flat or decreasing, whereas *V*_E_/*V*CO_2_ remains constant or decreases, and RER versus exercise time, which is flat or slowly increasing, and begins to increase more sharply; or the fraction of end-tidal O_2_ increases after registering as flat or decreasing, whereas the fraction of end-tidal CO_2_ remains constant or increases. The anaerobic threshold was expressed as *V*O_2_. The *V*_E_/*V*CO_2_ slope was calculated as the slope of the linear relationship between *V*_E_ and *V*CO_2_ from 1 min after the beginning of loaded exercise to the end of the isocapnic buffering period. All patients safely completed CPX at their maximum without angina pain or ischemic electrocardiographic changes.

### CR program

Patients in the exercise group attended exercise training as outpatients two or three times per week. Training sessions, which were performed under continuous electrocardiographic monitoring, were supervised by a cardiologist and a nurse. Each session was preceded by a 15 min warm-up followed by a 15 min cool-down. Exercise was performed for 20 min on a bicycle ergometer at the anaerobic threshold level as determined by the initial symptom-limited CPX. The workload was adjusted according to the CPX results every 3 months.

Patients attended education classes that were held in-hospital at the beginning of this program. These classes included lectures on coronary artery disease, secondary prevention, diet, smoking cessation, medication, and physical activities that were provided by physicians, physical therapists, nurses, and pharmacists. All patients also received individual counseling on exercise prescription, secondary prevention, and daily life activities from physicians and physical therapists once a week.

### Measurement of heart rate recovery for patients with AF

After achieving peak workload, all patients spent at least 5 min in a cool-down period during CPX. The value for heart rate recovery (HRR) was defined as a reduction in heart rate from the rate at peak exercise to the rate at 1 min after cessation of exercise. Heart rate was calculated as a mean of the last ten beats at each point.

### Statistical analysis

All statistical analyses were performed with EZR, which is a graphical user interface for *R*. More precisely, EZR is a modified version of *R* commander designed to add statistical functions that are frequently used in biostatistics. Continuous variables that followed a normal distribution are shown as mean ± standard deviation and the paired *t *test was used for comparison. Variables that did not follow a normal distribution are shown as the median and were compared using the Wilcoxon rank-sum test. We estimated unadjusted univariate linear regression coefficients between various variables with the % change in peak *V*O_2_ as the dependent variable. Laboratory variables, echocardiographic variables, and CPX parameters were the independent variables. Multiple linear regression analysis was carried out to assess the independent factors affecting the % change in peak *V*O_2_. A two-sided *p* value < 0.05 was considered significant.

## Results

### Clinical characteristics

Table 1 shows the clinical characteristics of the patients. A total of 84 patients (51 men, mean age: 68.5 ± 10.2 years) were examined. All patients suffered from persistent AF of New York Heart Association functional classes I (57%) and II (43%). Most (90%) patients had long-standing persistent AF. With regard to the cardiovascular background, the percentage of patients with ischemic heart disease was 36%. Beta blockers, renin–angiotensin system blockers, and diuretics were prescribed to most of the patients. There were no patients who prescribed sodium-glucose cotransporter two inhibitors which might reduce sympathetic nerve activity. All patients were medicated with direct oral anticoagulants. Initial medication for all patients did not change during 5 months. The mean attendance time for CR during 5 months was 44 ± 10 sessions (2.2 ± 0.8 sessions per week).

**Table 1:** Baseline patients’ characteristics

	*N* = 84
Age, years	68.5 ± 10.2
Male	60%
BMI (kg/m^2^)	25.0 ± 3.3
Comorbidies	
Hypertension	63%
Diabetes mellitus	33%
Dilated cardiomyopathy	45%
Coronary disease	36%
Medication	
Beta-blocker	90%
Digitalis	5%
RAS blockers	88%
Diuretics	95%
Aldosterone antagonist	48%
CCB	8%
DOAC	100%

Values are mean ± standard deviation or number (%)

*BMI* body mass index, *RAS* renin–angiotensin system, *CCB* calcium channel blocker, *DOAC* direct oral anticoagulant

### Effects of CR on laboratory and echocardiographic values

Table 2 shows laboratory and echocardiographic values at baseline and 5 months after CR. Plasma low-density lipoprotein cholesterol and B-type natriuretic peptide (BNP) levels were significantly improved after CR compared with baseline (*p* = 0.03, *p* < 0.01, respectively). With regard to echocardiographic values, only left atrial dimension was significantly improved after CR compared with baseline (*p* < 0.01), but the left ventricular ejection fraction did not improve.

**Table 2:** Laboratory values and echocardiographic parameters at baseline and at 5 month CR

	Baseline	5 month CR	*p* value
Laboratory values			
Sodium (mEq/L)	138 ± 13	138 ± 10	n.s
LDL cholesterol (mg/dL)	124 ± 10	119 ± 8	*p* = 0.03
Hemoglobin (g/dL)	13.2 ± 2.3	14.2 ± 5.3	n.s
eGFR (ml/min/1.73m^2^)	65 ± 6.5	66 ± 8.5	n.s
BNP (pg/mL)	352.7 ± 126.7	327.7 ± 100.7	*p* < 0.01
Echocardiographic values			
LAD (mm)	49.5 ± 10.1	45.5 ± 12.1	*p* < 0.01
LVDs (mm)	50 ± 10	49 ± 8	n.s
E/e’	18.6 ± 5.1	17.8 ± 4.3	n.s
LVEF (%)	45 ± 10	46 ± 9	n.s

Values are mean ± standard deviation

*LDL* low-density lipoprotein, *eGFR* estimated glomerular filtration rate, *BNP* B-type natriuretic peptide, *LAD* left atrial dimension, *LVDs* left ventricular end-systolic dimension, *E/e’* early diastolic transmitral inflow velocity/early diastolic mitral annulus velocity, *LVEF* left ventricular ejection fraction, *CR* cardiac rehabilitation

### Effects of CR on CPX parameters

Table 3 shows CPX parameters at baseline and 5 months after CR. The peak RER, peak heart rate, peak workload, peak *V*O_2_, and HRR, and were significantly elevated after CR compared with baseline (*p* = 0.03, *p* < 0.01, *p* = 0.03, *p* < 0.01, and *p* < 0.01, respectively). Resting heart rate and the *V*_E_/*V*CO_2_ slope were significantly reduced after CR compared with baseline (*p* < 0.01, *p* < 0.01, respectively). Parameters of blood pressure were not significantly changed 5 months after CR.

**Table 3:** Parameters of the cardiopulmonary exercise test before and after cardiac rehabilitation

	Baseline	5 month CR	*p* value
Peak RER	1.32 ± 0.16	1.36 ± 0.22	*p* = 0.03
Resting heart rate (bpm)	82 ± 13	78 ± 17	*p* < 0.01
Resting SBP (mmHg)	117 ± 26	120 ± 27	n.s
Resting DBP (mmHg)	73 ± 14	74 ± 16	n.s
Peak heart rate (bpm)	149 ± 19	154 ± 16	*p* < 0.01
Peak SBP (mmHg)	155 ± 27	157 ± 24	n.s
Peak DBP (mmHg)	90 ± 14	89 ± 13	n.s
Peak work load (watt)	65 ± 15	68 ± 14	*p* = 0.03
Peak *V*O_2_ (ml/kg/min)	16.2 ± 3.5	19.2 ± 4.2	*p* < 0.01
*V*E/*V*CO_2_ slope (%)	30.6 ± 5.1	27.5 ± 6.4	*p* < 0.01
Heart rate recovery	10 ± 4	14 ± 5	*p* < 0.01

Values are mean ± standard deviation

*RER* respiratory exchange ratio, *SBP* systolic blood pressure, *DBP* diastolic blood pressure, *V*O_2_ oxygen uptake, *V*_*E*_*/V*CO_2_ minute ventilation/carbon dioxide output

### Comparison of the % change in all values with that of peak *V*_O2_

**Fig.1:**
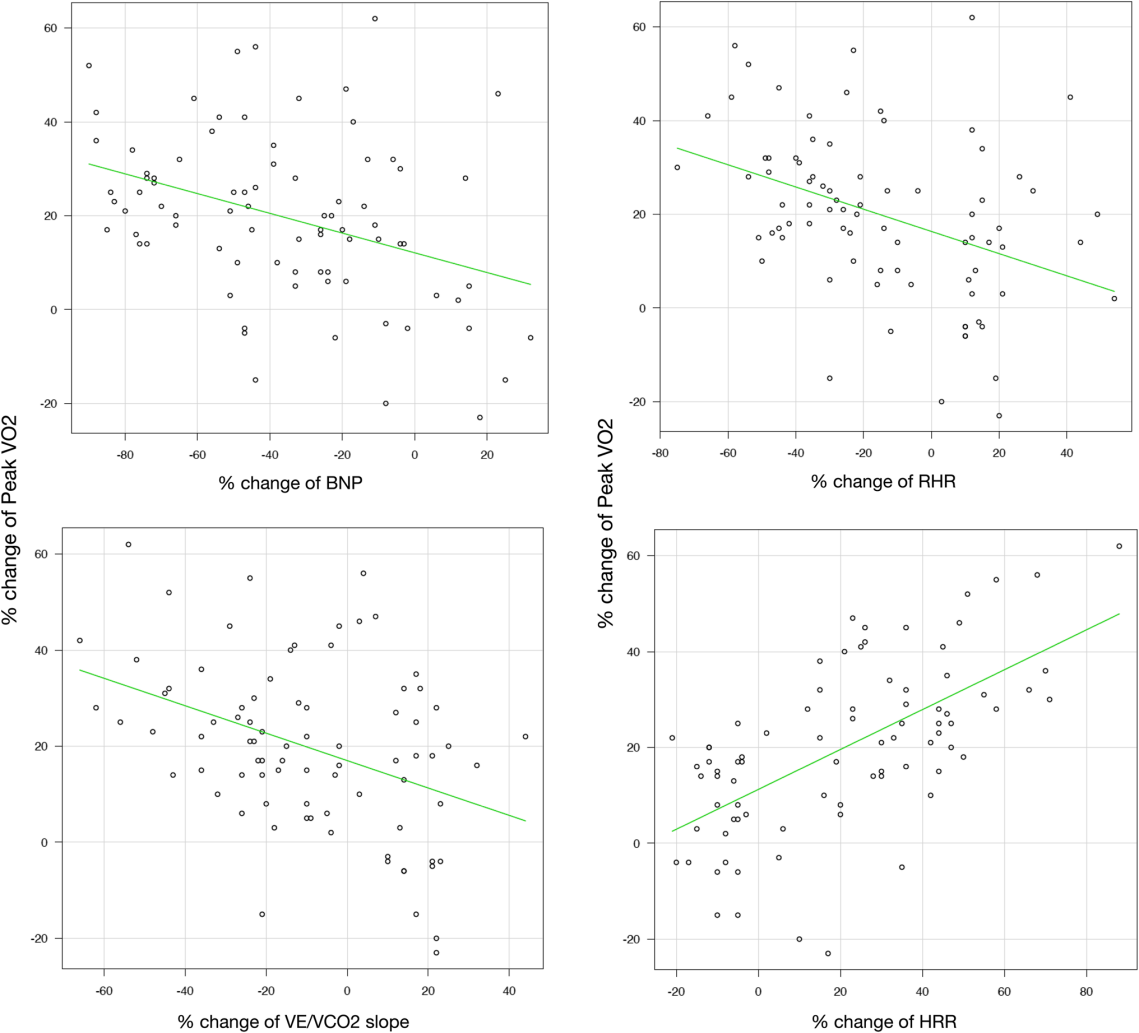
Correlative relationships between the percentage change in peak VO2 and that of plasma BNP levels, RHR, the VE/VCO2 slope, and HRR. *BNP* B-type natriuretic peptide, *RHR* resting heart rate, *VE/VCO2* minute ventilation/carbon dioxide output, *HRR* heart rate recovery

We investigated the contributions to peak *V*O_2_ between baseline and 5 months after CR. The % change is where designated values were compared between baseline and after CR. The % change in peak *V*O_2_ was significantly correlated with that of plasma BNP levels, resting heart rate, the *V*_E_/*V*CO_2_ slope, and HRR in univariate linear regression analysis (Fig. 1). We applied multivariate linear regression analysis to determine which values were independently correlated. Table 4 shows that the % change in HRR was independently correlated with that of peak *V*O_2_ (*p* = 0.02).

**Table 4:** Univariate and multivariate linear regression analyses for identifying independent factors associated with the rate of % change in peak *V*O_2_

% change of *X* (*X*post – *X*pre/*X*pre)	Univariate		Multivariate	
	Unstandardized coefficient B	*p* value	Unstandardized coefficient B	*p* value
BNP	− 0.31	0.03	− 0.22	0.36
Resting heart rate	− 0.36	0.02	− 0.18	0.25
*V*_E_/*V*CO_2_ slope	0.55	< 0.01	0.32	0.15
Heart rate recovery	0.43	< 0.01	0.22	0.02

*V*O_2_ oxygen uptake, *BNP* B-type natriuretic peptide, *V*_*E*_*/V*CO_2_ minute ventilation/carbon dioxide output

## Discussion

In this retrospective study, we found that HRR is significantly associated with recovery of exercise tolerance in patients with HF and AF. To the best of our knowledge, it has not previously been shown to be associated with AF.

In patients with HF and AF, abnormal neurohormonal control may explain the mechanism of advancement of disease. The amount of circulating catecholamines in patients is related to the prognosis [15]. Studies of the pathophysiology of autonomic control in the failing heart that analyzed heart rate variability (HRV) showed progressive abnormalities in the power spectral profile of patients [16, 17]. Buchheit et al. reported that the relations between HRV, HRR, and parasympathetic reactivation were low [18]. A lack of these relationships is due to many factors, such as sympathetic activity, ventilation, and environmental effects, which might interfere with parasympathetic outflow during the post-exercise period, and probably to a large extent, during resting conditions. For these reasons, resting HRV is reliable, whereas the reliability of HRR is considered satisfactory [19]. Recently, Astolfi et al. reported that increased HRR after the 6 min walk test was positively correlated with the 6 min walk test distance in patients after acute coronary syndrome [20]. These authors also found that HRR appeared to be more sensitive than post-exercise HRV analysis for monitoring functional and autonomic improvement after acute coronary syndrome. Therefore, HRR after symptom-limited CPX in our study was a good candidate for evaluating parasympathetic reactivation. To date, a lot of evidence has shown that autonomic dysfunction and decreased parasympathetic function play a significant role in development of AF [21–24]. Maddox et al. reported that impaired HRR was significantly associated with a higher likelihood of new-onset AF, independent of patients and clinical factors [25]. These findings support the concept of the autonomic nervous system as a potential mediator of AF.

Recently, Sydo et al. showed that impaired HRR was associated with a number of well-established cardiovascular risk factors, including diabetes mellitus, hypertension, current smoking, and chronic kidney disease [26]. Increased parasympathetic activity is a protective mechanism of exercise training implemented during CR, in addition to pharmacological treatment and invasive non-pharmacological interventions [27]. In a pioneering study, Coats et al. showed a significant shift towards parasympathetic modulation of autonomic control after a 6 month period of training [28]. Myers et al. also investigated the association between exercise tolerance and improvement in HRR in post-ACS patients after 8 weeks of a rehabilitation program [29]. These authors evaluated exercise tolerance of patients by peak *V*O_2_ under CPX, as performed in our study.

The report from Myers et al. [29] showed better HRR improvement in HF patients with sinus rhythm than our patients with AF although training period was different. The parasympathetic portion decrease sinus node automaticity and slows atrioventricular node conduction in patients with sinus rhythm. In patients with AF, however, parasympathetic effect is limited to atrioventricular node. This might be the reason why there is difference between two studies.

In the end of the discussion, we have to mention the sympathetic modulation to HRR. Previous studies have shown that early HRR after exercise is mainly a function of parasympathetic reactivation, with the sympathetic withdrawal becoming important later in recovery. A delayed HRR is, therefore, considered a measure of autonomic imbalance and may be an indicator of a reduction in parasympathetic tone or an exaggerated sympathetic activation [30–32]. According to those findings our data of HRR, which indicate very early parameter after exercise, was modulated mainly by parasympathetic tone. However, we did not check the change in adrenaline that would show evidence of enhanced parasympathetic nerve system and reduced sympathetic nerve system activity. Further examination will be needed to clarify what extent the sympathetic improvement contribute to the recovery of exercise tolerance of patients with HF and AF after CR.

### Study limitations

This study has several important limitations. First, this was a retrospective observational study although we enrolled consecutive patients meeting the inclusion and exclusion criteria. Second, we did not have enough peripheral parameters other than those of autonomic nerve system, such as skeletal muscle function, vascular endothelial function. We cannot exclude that those factors might influence the changes of peak *V*O_2_. Third, we cannot comment on the value of HRR as measured over different time points, such as 2, 3 or 5 min after cessation of exercise, although we identified HRR as the change during the first minute of the active recovery period. Forth, we did not have data of HRV which is one of parameters of autonomic function, although we discussed the relations between HRR and HRV in the discussion section. Fifth, we did not design our study to compare patients between heart failure with reduced ejection fraction and with preserved ejection fraction. We did not have any results of differences between two groups.

## Conclusions

This study provides new evidence of the association between improved HRR and improved exercise tolerance in patients with HF and AF. Our findings have interest from a clinical point of view because HRR is easily measurable and was found useful. Future research to validate these findings in other populations will reveal the mechanistic links between HRR and exercise tolerance.
